# Tubercle Bacilli in Sputum

**Published:** 1911-05-06

**Authors:** 


### TUBERCLE BACILLI IN SPUTUM.

The difficulty of detecting tubercle bacilli in the sputa of certain patients known to be suffering from pulmonary tuberculosis is familiar to all, and not a few laboratory methods of concentrating the bacilli from a bulk of sputum have been advocated from time to time. Two of these seem to be of particular value—namely, the antiformin method of Uhlenhuth and Xylander, and the chloroform method of Loeffler.

Antiformin consists of a mixture of equal parts of sodium hypochlorite and sodium hydrate, specially prepared; the sputum thrown into a 15 per cent. solution of this, having been rendered homogeneous, is centrifugalised in a machine revolving at a high rate; the supernatant fluid having then been poured off, the deposit is shaken up with physiological salt solution and then centrifugalised again. The excess of salt solution having been poured off, the concentrated residue is utilised for the making of films, which are dried, fixed, and stained by Ziehl-Neelsen method in the ordinary way. The process occupies a relatively short time, and it is efficacious.

This is a modification of the antiformin method, and it is carried out as follows: A quantity of sputum is mixed with an equal amount of 50 per cent. antiformin, and the mixture is heated until it has become clear and liquid. Ten c.c. of the latter are then pipetted off into a separate receiver, and 5 c.c. of a 10 per cent. solution of chloroform in alcohol are added. This mixture is shaken well together, and then centrifugalised at a high rate for a quarter of an hour. The chloroform will then be found to be at the bottom of the tube, separated from the supernatant fluid by an opaque line of variable thickness. As much as possible of the superficial fluid is removed with a pipette, and the opaque layer on the surface of the chloroform carefully removed on to a slide upon which a film is made, dried, fixed, and stained in the ordinary way.

